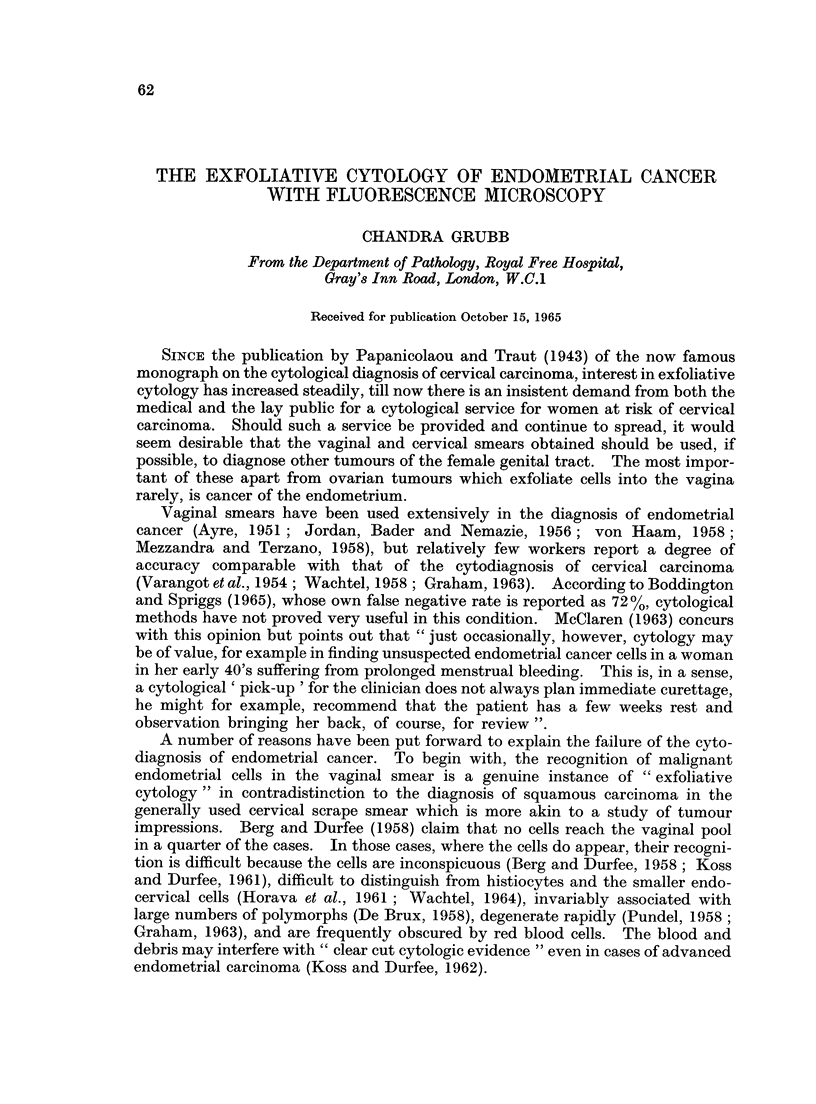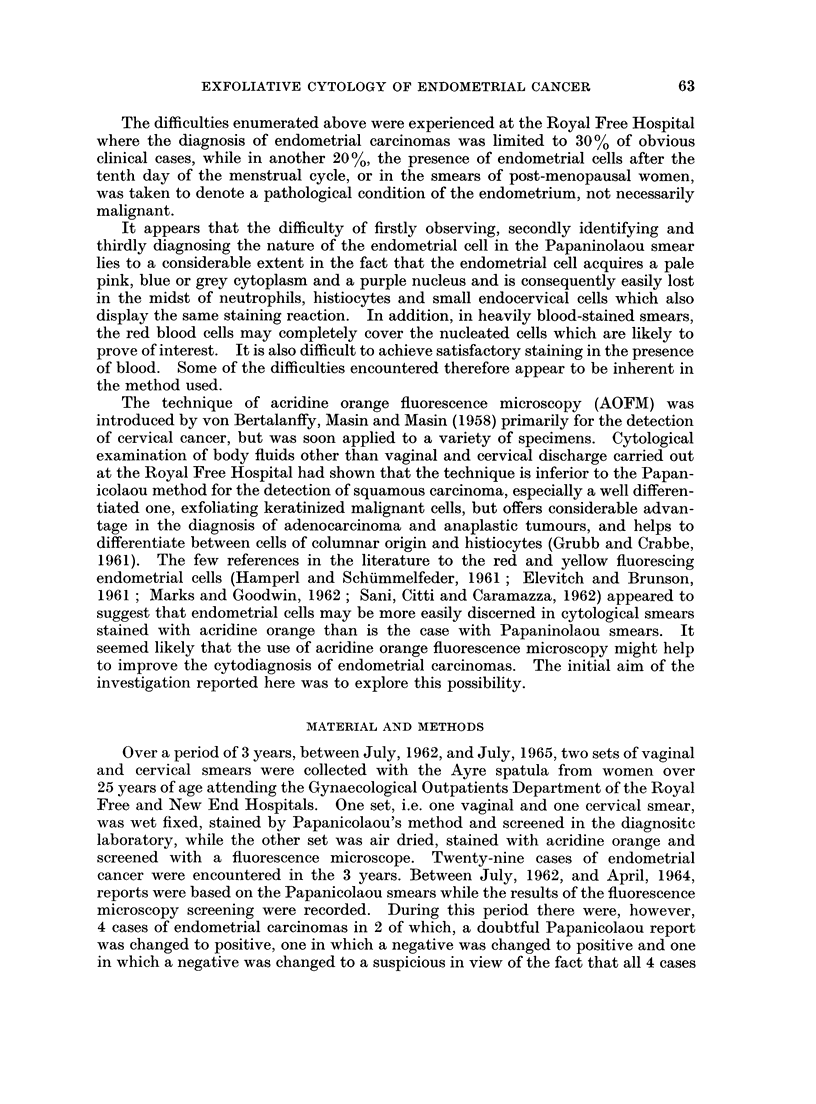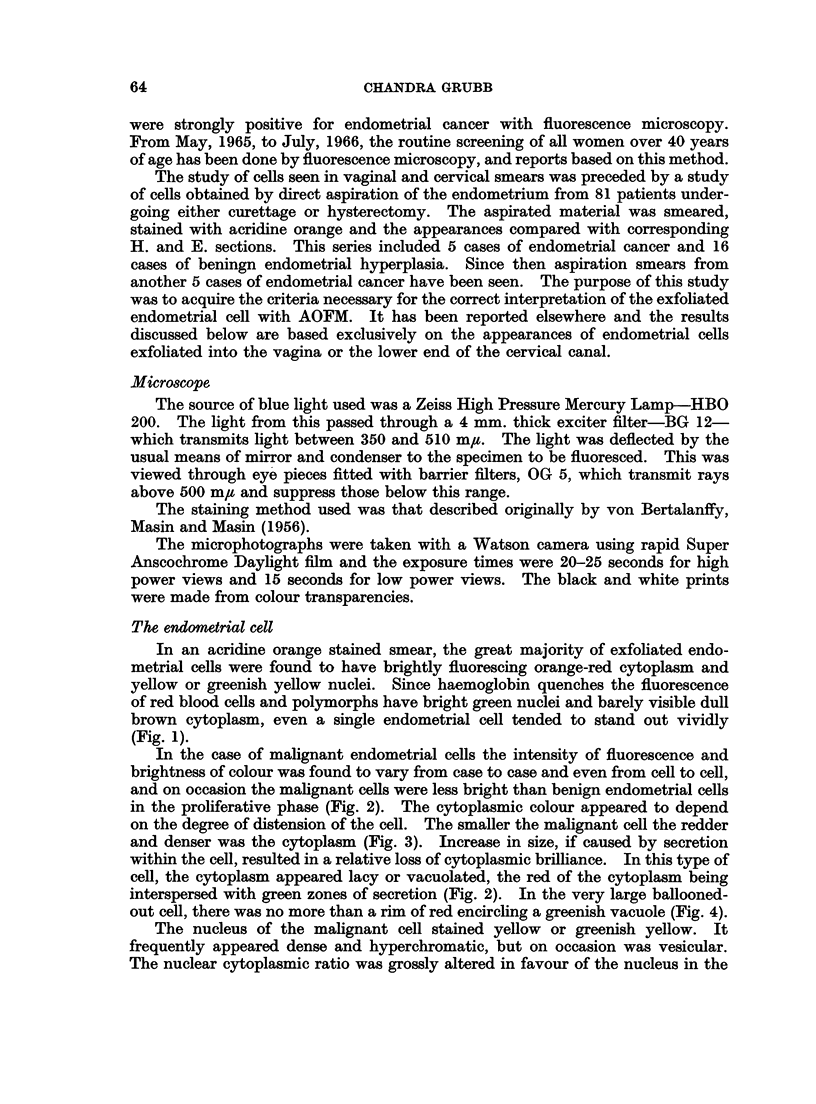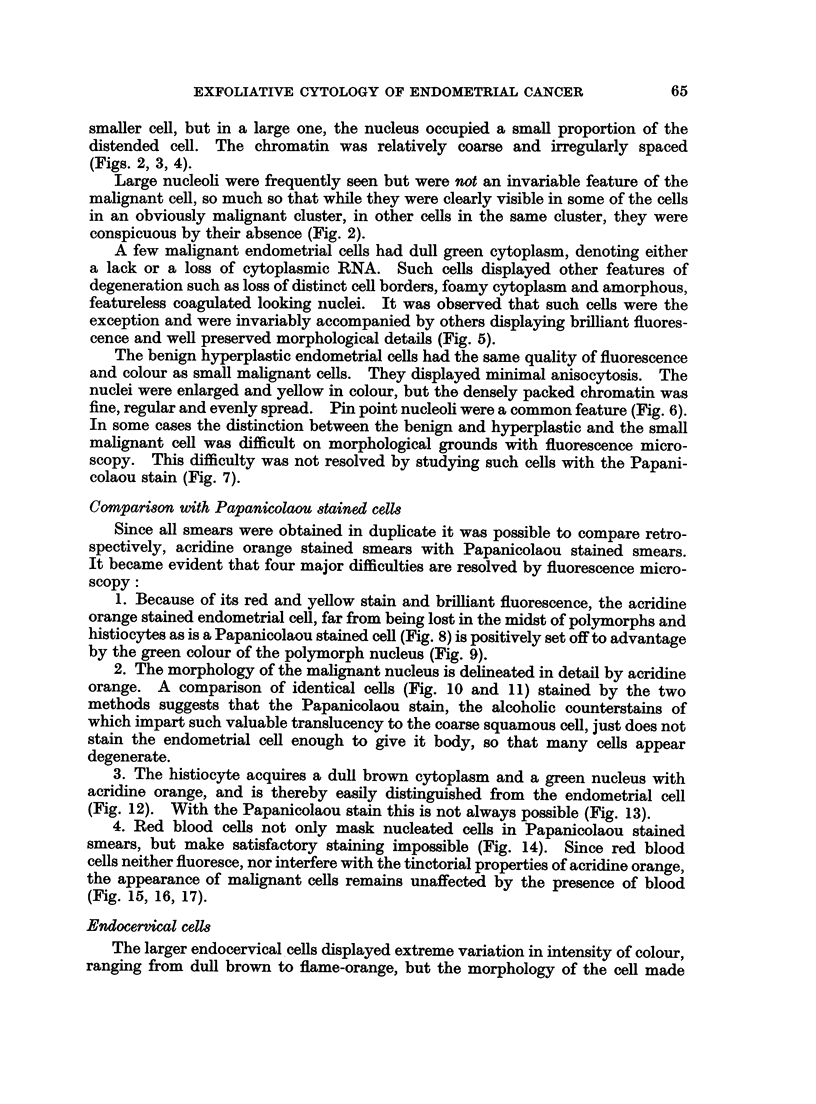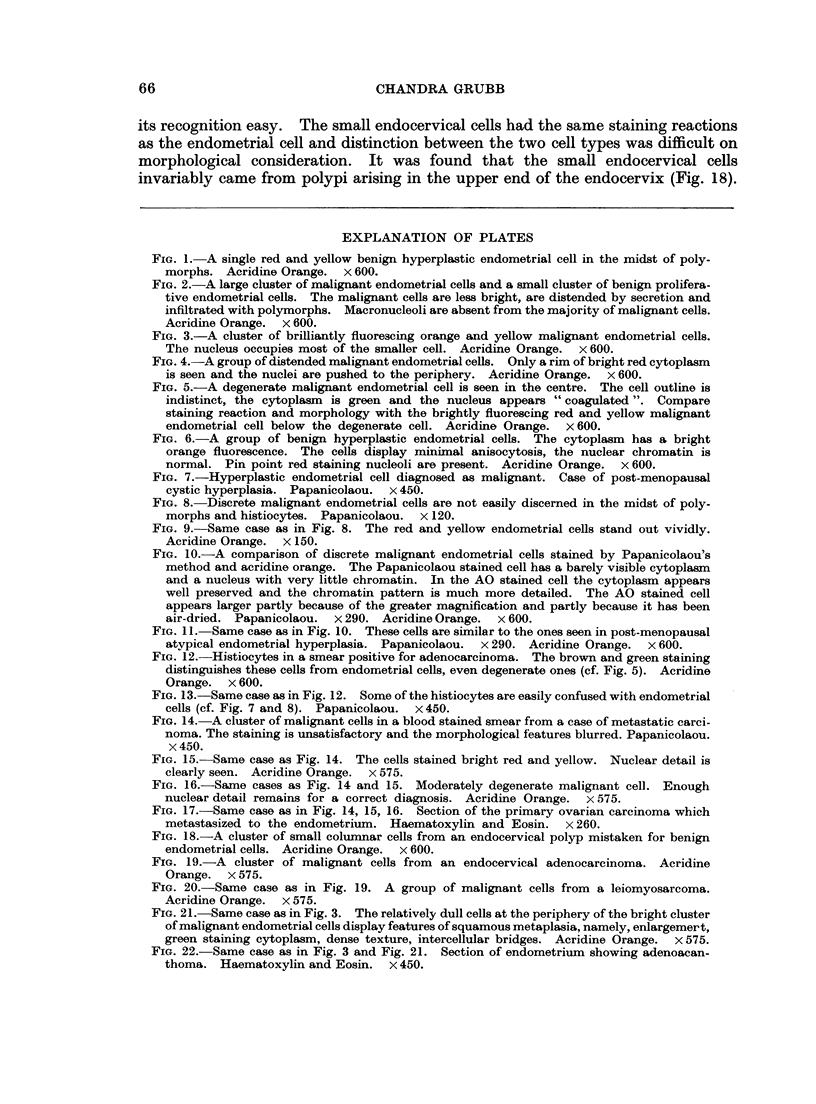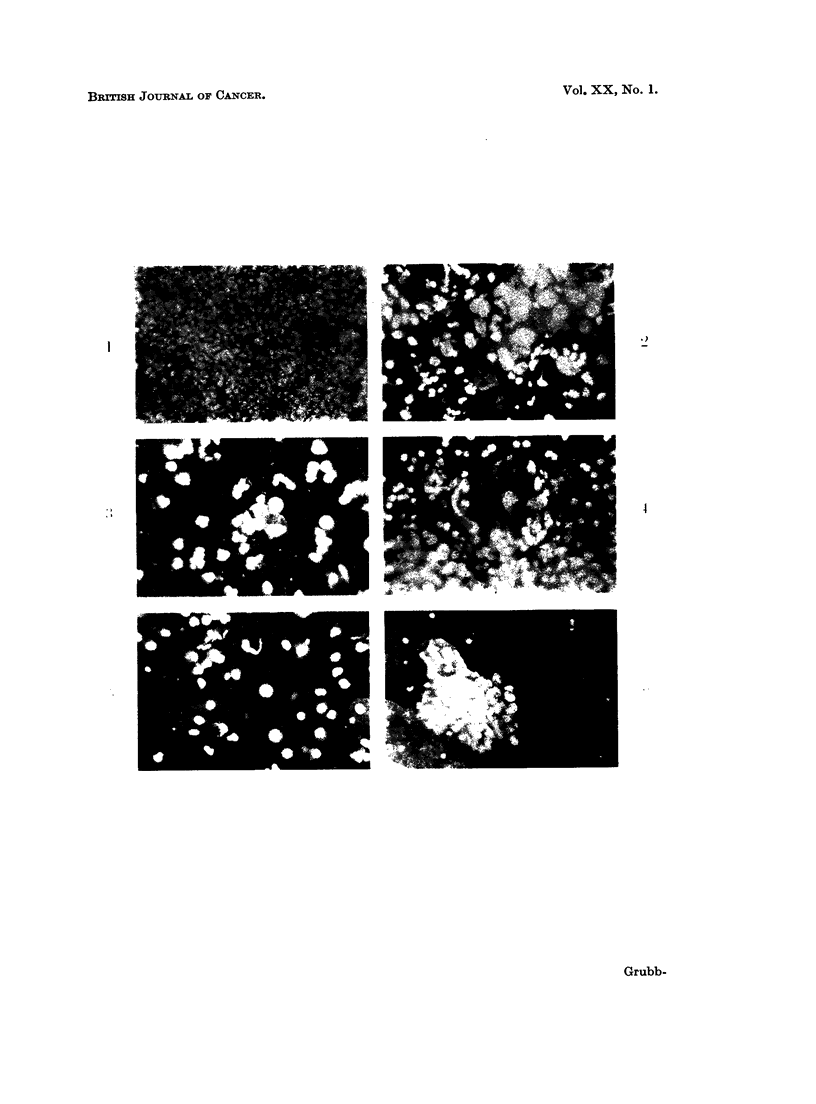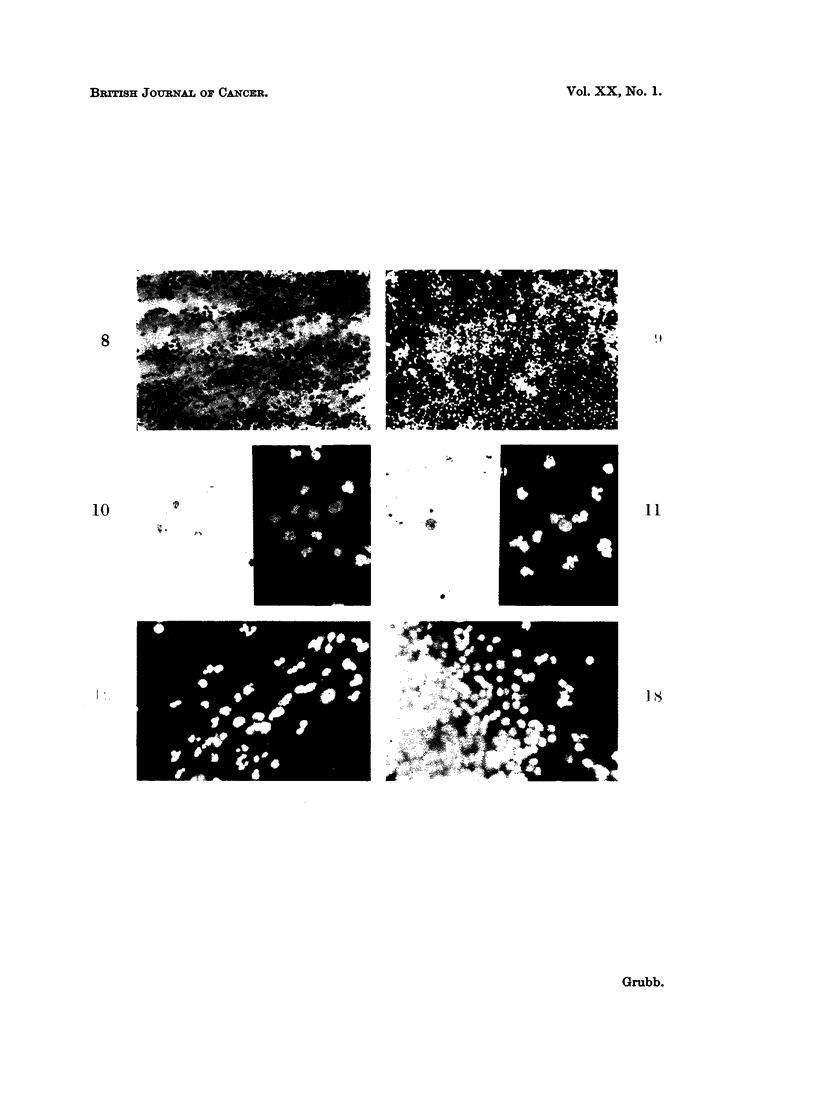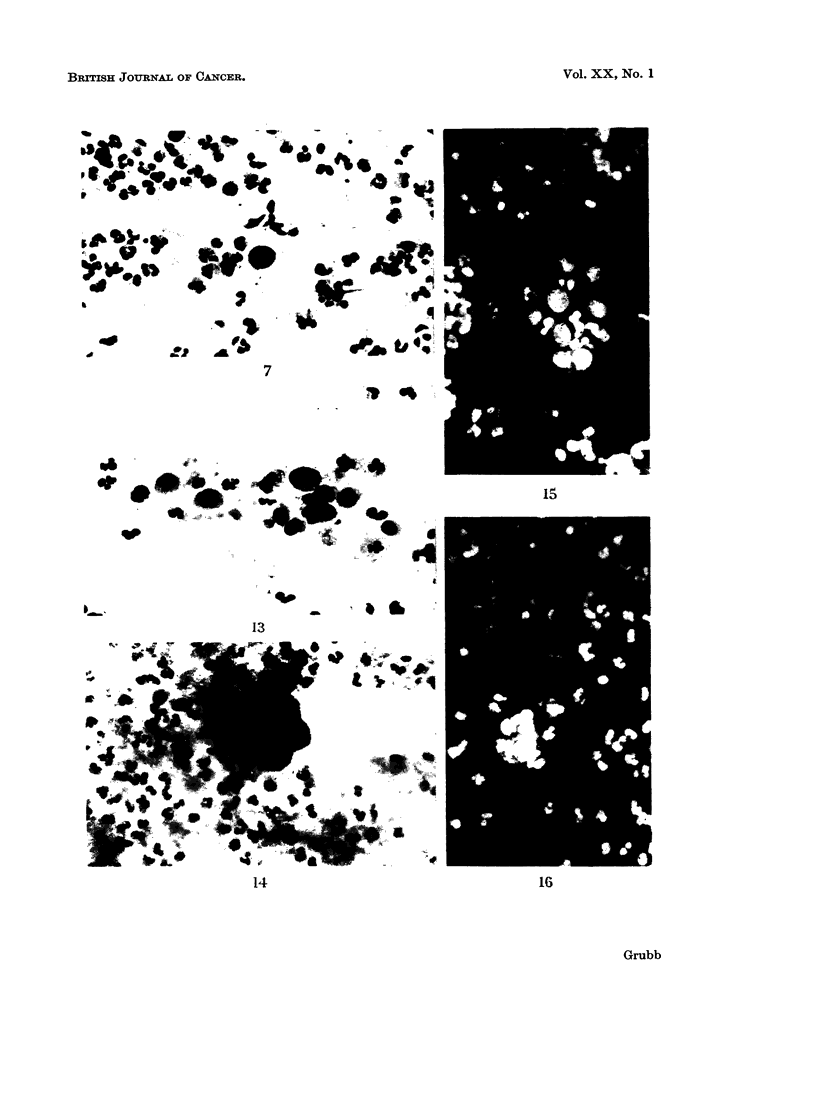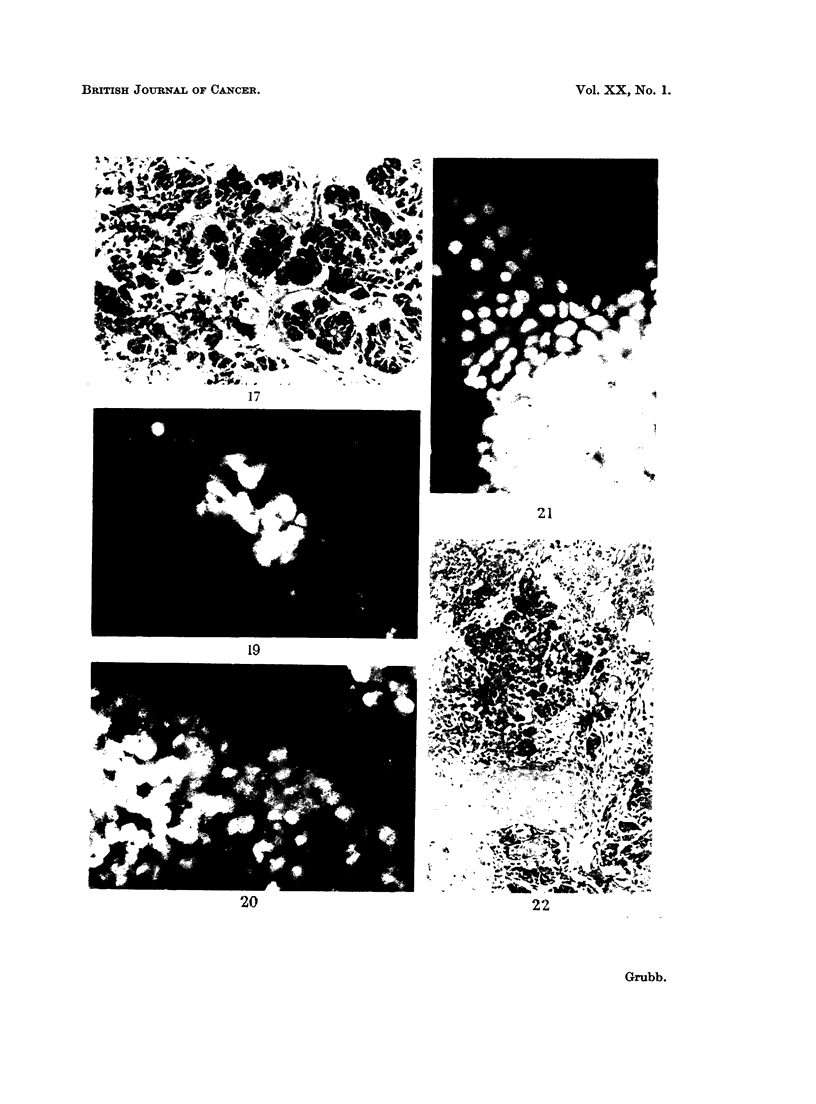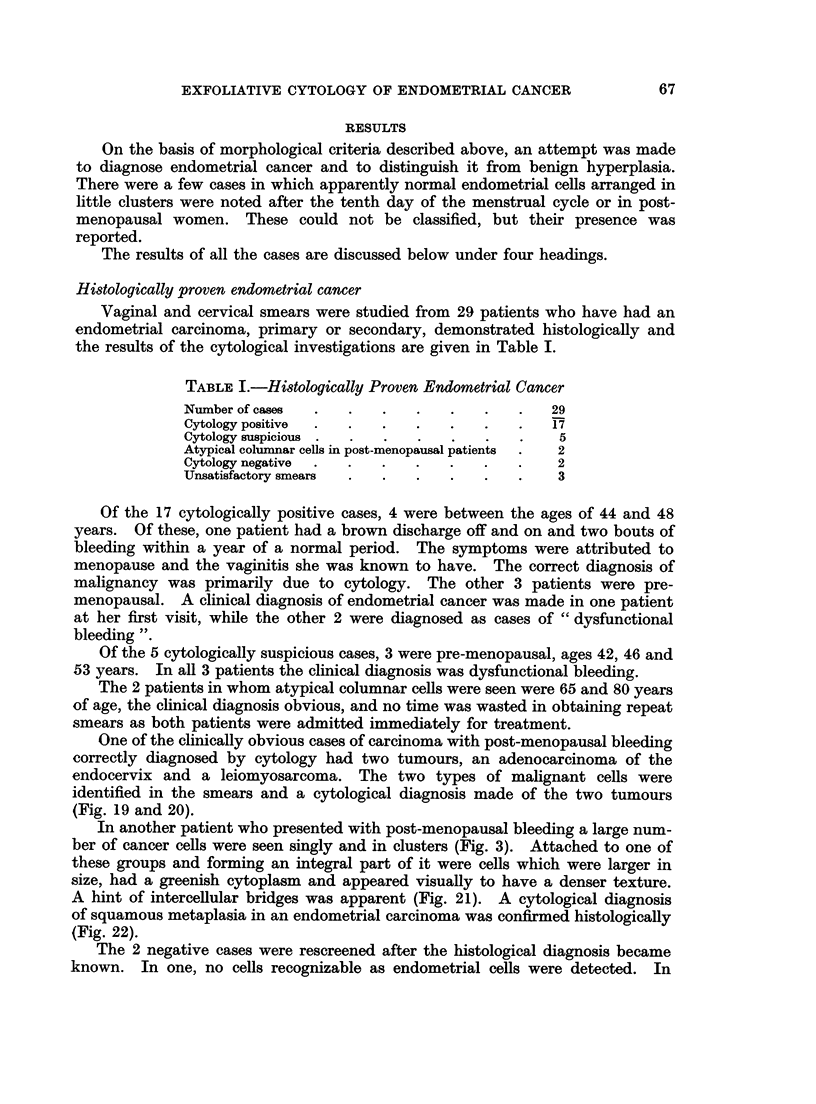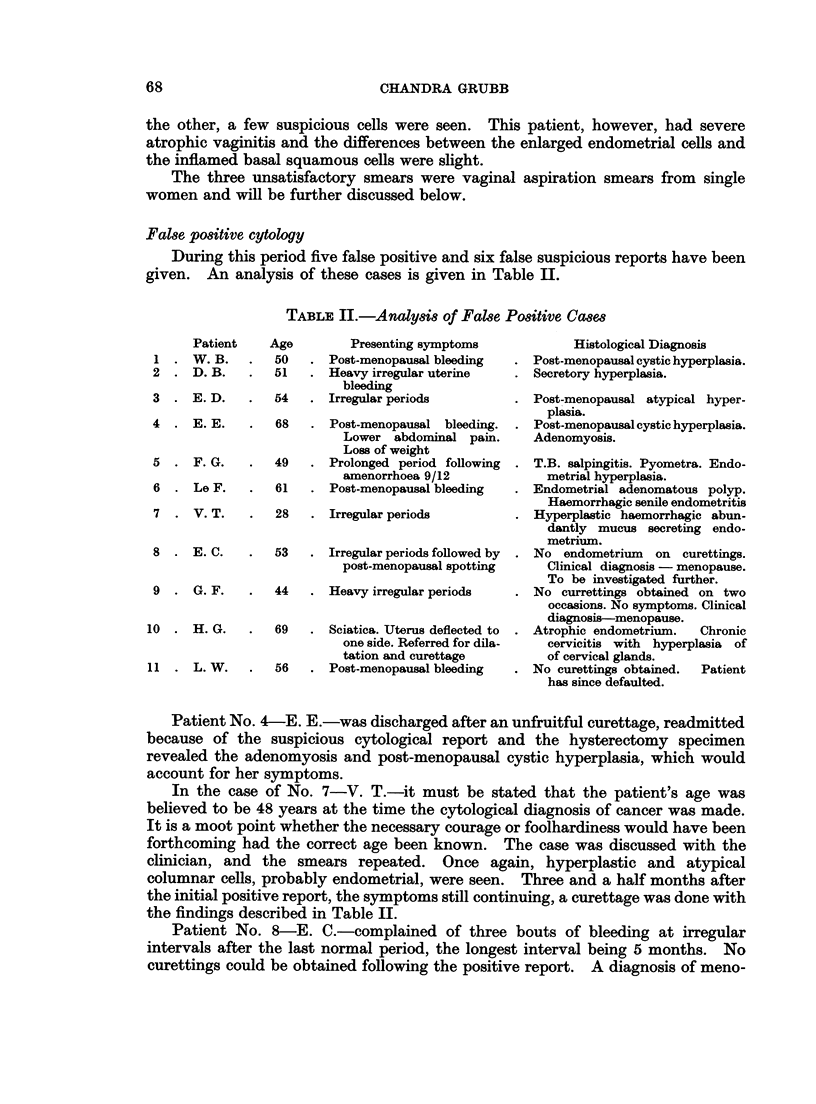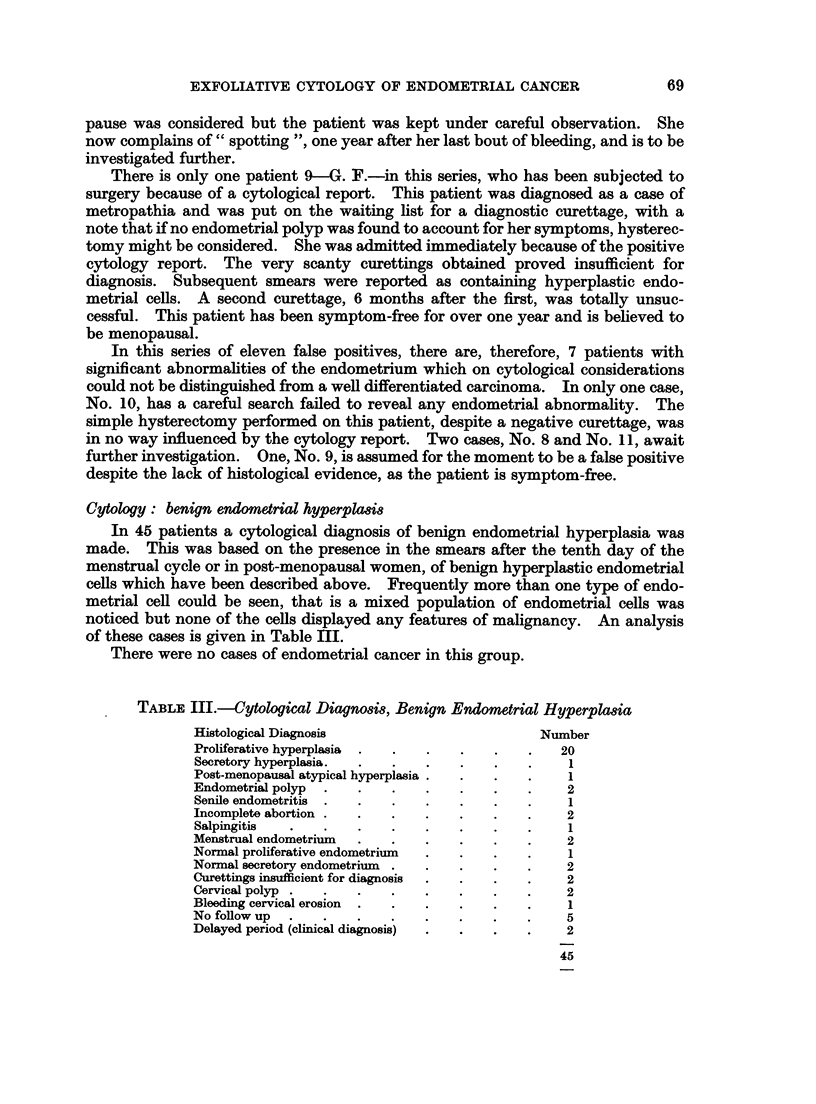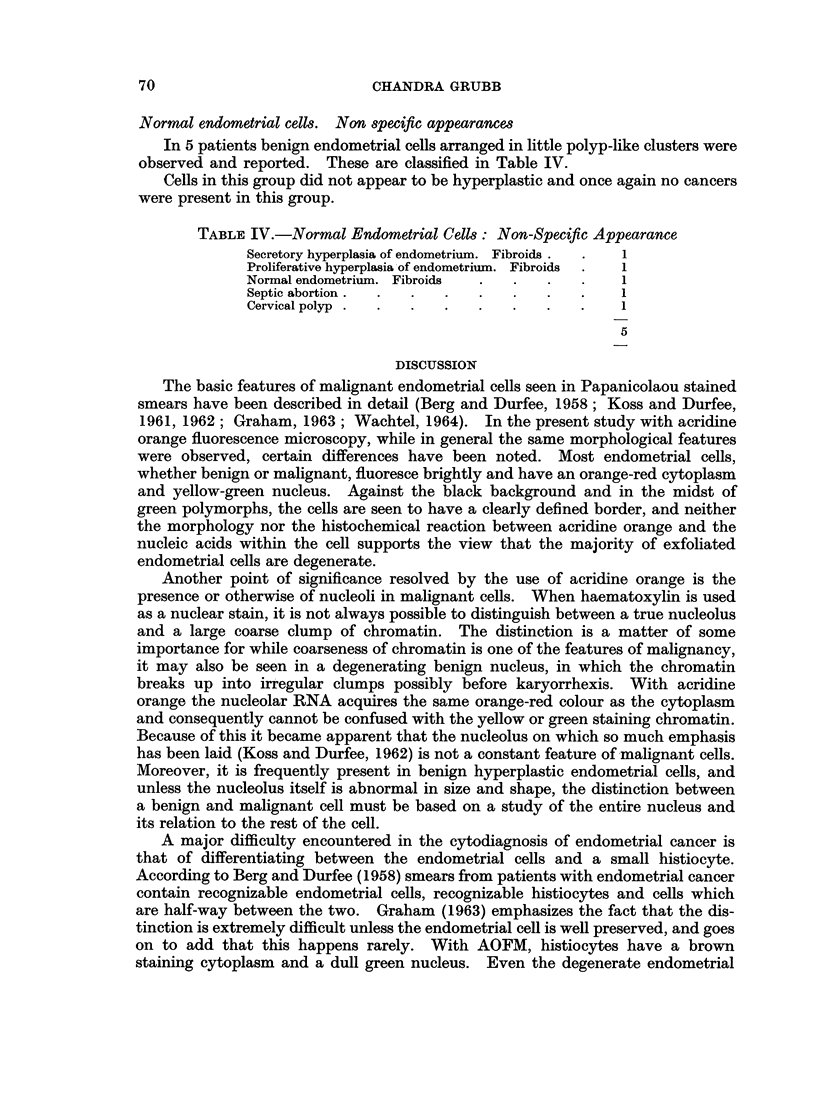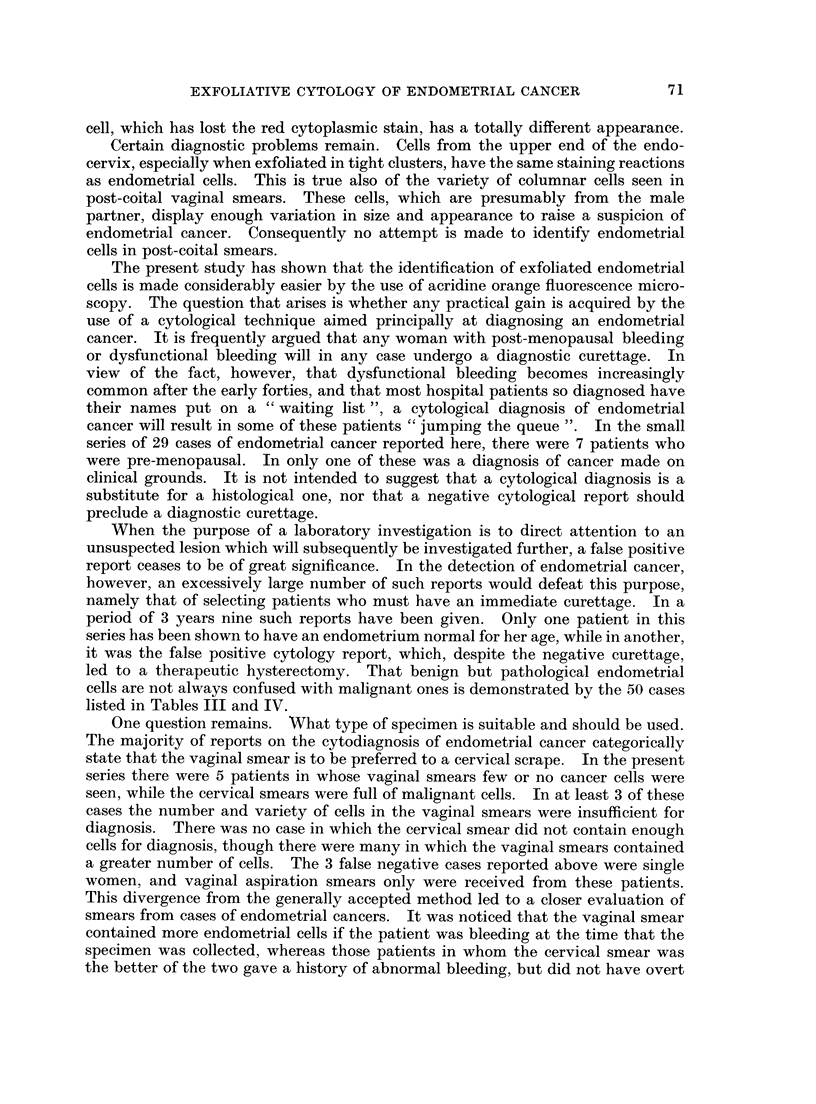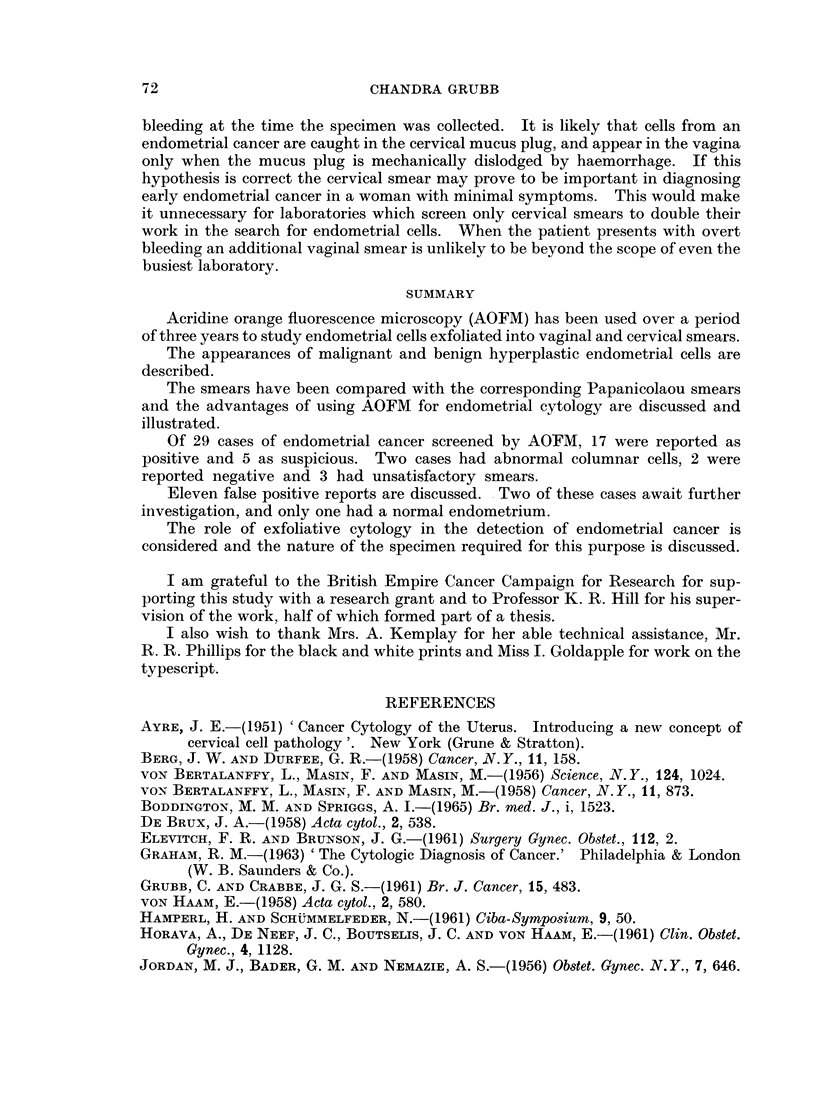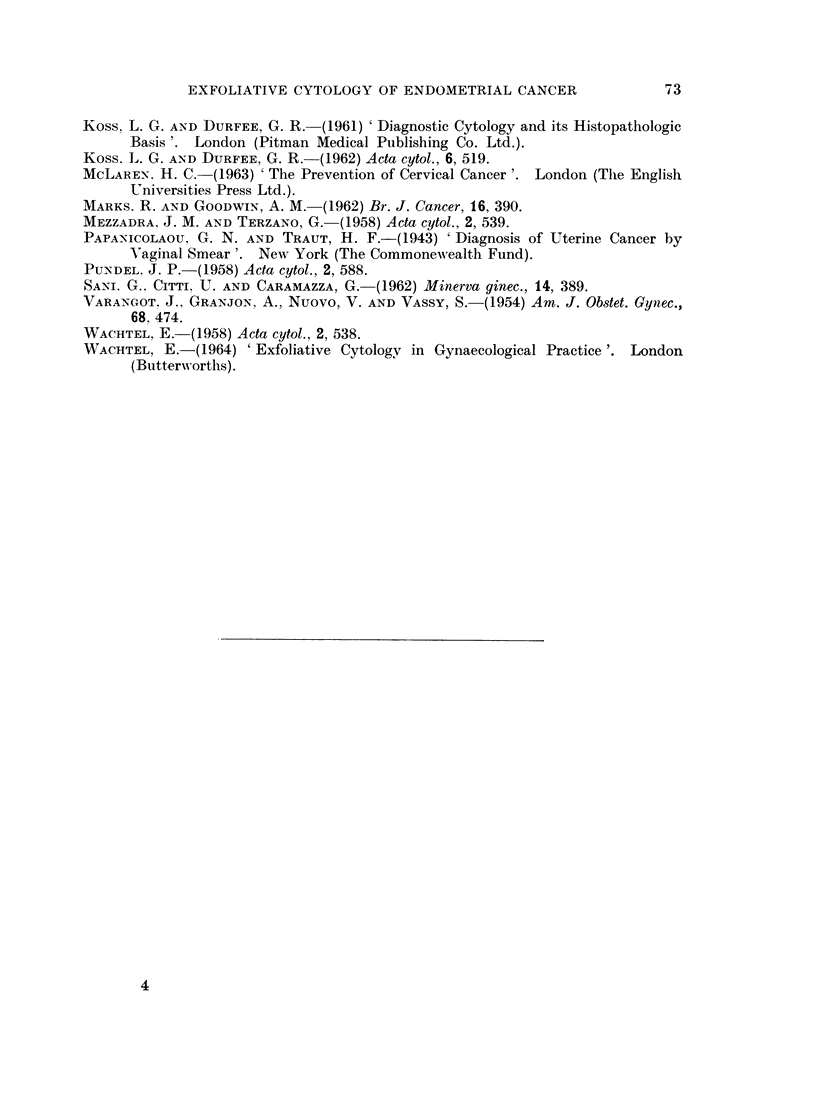# The exfoliative cytology of endometrial cancer with fluorescence microscopy.

**DOI:** 10.1038/bjc.1966.8

**Published:** 1966-03

**Authors:** C. Grubb

## Abstract

**Images:**


### THE EXFOLIATIVE CYTOLOGY OF ENDOMETRIAL CANCER WITH FLUORESCENCE MICROSCOPY

CHANDRA GRUBB

*From the Department of Pathology, Royal Free Hospital, Gray's Inn Road, London, W.C.*1

Received for publication October 15, 1965

SINCE the publication by Papanicolaou and Traut (1943) of the now famous monograph on the cytological diagnosis of cervical carcinoma, interest in exfoliative cytology has increased steadily, till now there is an insistent demand from both the medical and the lay public for a cytological service for women at risk of cervical carcinoma. Should such a service be provided and continue to spread, it would seem desirable that the vaginal and cervical smears obtained should be used, if possible, to diagnose other tumours of the female genital tract. The most important of these apart from ovarian tumours which exfoliate cells into the vagina rarely, is cancer of the endometrium.

Vaginal smears have been used extensively in the diagnosis of endometrial cancer (Ayre, 1951 ; Jordan, Bader and Nemazie, 1956 ; von Haam, 1958 ; Mezzandra and Terzano, 1958), but relatively few workers report a degree of accuracy comparable with that of the cytodiagnosis of cervical carcinoma (Varangot *et al.*, 1954 ; Wachtel, 1958 ; Graham, 1963). According to Boddington and Spriggs (1965), whose own false negative rate is reported as 72%, cytological methods have not proved very useful in this condition. McClaren (1963) concurs with this opinion but points out that " just occasionally, however, cytology may be of value, for example in finding unsuspected endometrial cancer cells in a woman in her early 40's suffering from prolonged menstrual bleeding. This is, in a sense, a cytological ' pick-up ' for the clinician does not always plan immediate curettage, he might for example, recommend that the patient has a few weeks rest and observation bringing her back, of course, for review ".

A number of reasons have been put forward to explain the failure of the cytodiagnosis of endometrial cancer. To begin with, the recognition of malignant endometrial cells in the vaginal smear is a genuine instance of " exfoliative cytology " in contradistinction to the diagnosis of squamous carcinoma in the generally used cervical scrape smear which is more akin to a study of tumour impressions. Berg and Durfee (1958) claim that no cells reach the vaginal pool in a quarter of the cases. In those cases, where the cells do appear, their recognition is difficult because the cells are inconspicuous (Berg and Durfee, 1958 ; Koss and Durfee, 1961), difficult to distinguish from histiocytes and the smaller endocervical cells (Horava *et al.*, 1961 ; Wachtel, 1964), invariably associated with large numbers of polymorphs (De Brux, 1958), degenerate rapidly (Pundel, 1958 ; Graham, 1963), and are frequently obscured by red blood cells. The blood and debris may interfere with " clear cut cytologic evidence " even in cases of advanced endometrial carcinoma (Koss and Durfee, 1962).

The difficulties enumerated above were experienced at the Royal Free Hospital where the diagnosis of endometrial carcinomas was limited to 30% of obvious clinical cases, while in another 20%, the presence of endometrial cells after the tenth day of the menstrual cycle, or in the smears of post-menopausal women, was taken to denote a pathological condition of the endometrium, not necessarily malignant.

It appears that the difficulty of firstly observing, secondly identifying and thirdly diagnosing the nature of the endometrial cell in the Papaninolaou smear lies to a considerable extent in the fact that the endometrial cell acquires a pale pink, blue or grey cytoplasm and a purple nucleus and is consequently easily lost in the midst of neutrophils, histiocytes and small endocervical cells which also display the same staining reaction. In addition, in heavily blood-stained smears, the red blood cells may completely cover the nucleated cells which are likely to prove of interest. It is also difficult to achieve satisfactory staining in the presence of blood. Some of the difficulties encountered therefore appear to be inherent in the method used.

The technique of acridine orange fluorescence microscopy (AOFM) was introduced by von Bertalanffy, Masin and Masin (1958) primarily for the detection of cervical cancer, but was soon applied to a variety of specimens. Cytological examination of body fluids other than vaginal and cervical discharge carried out at the Royal Free Hospital had shown that the technique is inferior to the Papanicolaou method for the detection of squamous carcinoma, especially a well differentiated one, exfoliating keratinized malignant cells, but offers considerable advantage in the diagnosis of adenocarcinoma and anaplastic tumours, and helps to differentiate between cells of columnar origin and histiocytes (Grubb and Crabbe, 1961). The few references in the literature to the red and yellow fluorescing endometrial cells (Hamperl and Schümmelfeder, 1961 ; Elevitch and Brunson, 1961 ; Marks and Goodwin, 1962 ; Sani, Citti and Caramazza, 1962) appeared to suggest that endometrial cells may be more easily discerned in cytological smears stained with acridine orange than is the case with Papaninolaou smears. It seemed likely that the use of acridine orange fluorescence microscopy might help to improve the cytodiagnosis of endometrial carcinomas. The initial aim of the investigation reported here was to explore this possibility.

### MATERIAL AND METHODS

Over a period of 3 years, between July, 1962, and July, 1965, two sets of vaginal and cervical smears were collected with the Ayre spatula from women over 25 years of age attending the Gynaecological Outpatients Department of the Royal Free and New End Hospitals. One set, i.e. one vaginal and one cervical smear, was wet fixed, stained by Papanicolaou's method and screened in the diagnositc laboratory, while the other set was air dried, stained with acridine orange and screened with a fluorescence microscope. Twenty-nine cases of endometrial cancer were encountered in the 3 years. Between July, 1962, and April, 1964, reports were based on the Papanicolaou smears while the results of the fluorescence microscopy screening were recorded. During this period there were, however, 4 cases of endometrial carcinomas in 2 of which, a doubtful Papanicolaou report was changed to positive, one in which a negative was changed to positive and one in which a negative was changed to a suspicious in view of the fact that all 4 cases were strongly positive for endometrial cancer with fluorescence microscopy. From May, 1965, to July, 1966, the routine screening of all women over 40 years of age has been done by fluorescence microscopy, and reports based on this method.

The study of cells seen in vaginal and cervical smears was preceded by a study of cells obtained by direct aspiration of the endometrium from 81 patients undergoing either curettage or hysterectomy. The aspirated material was smeared, stained with acridine orange and the appearances compared with corresponding H. and E. sections. This series included 5 cases of endometrial cancer and 16 cases of beningn endometrial hyperplasia. Since then aspiration smears from another 5 cases of endometrial cancer have been seen. The purpose of this study was to acquire the criteria necessary for the correct interpretation of the exfoliated endometrial cell with AOFM. It has been reported elsewhere and the results discussed below are based exclusively on the appearances of endometrial cells exfoliated into the vagina or the lower end of the cervical canal.

*Microscope*

The source of blue light used was a Zeiss High Pressure Mercury Lamp—HBO 200. The light from this passed through a 4 mm. thick exciter filter—BG 12—which transmits light between 350 and 510 m$\mu$. The light was deflected by the usual means of mirror and condenser to the specimen to be fluoresced. This was viewed through eye pieces fitted with barrier filters, OG 5, which transmit rays above 500 m$\mu$ and suppress those below this range.

The staining method used was that described originally by von Bertalanffy, Masin and Masin (1956).

The microphotographs were taken with a Watson camera using rapid Super Anscochrome Daylight film and the exposure times were 20–25 seconds for high power views and 15 seconds for low power views. The black and white prints were made from colour transparencies.

*The endometrial cell*

In an acridine orange stained smear, the great majority of exfoliated endometrial cells were found to have brightly fluorescing orange-red cytoplasm and yellow or greenish yellow nuclei. Since haemoglobin quenches the fluorescence of red blood cells and polymorphs have bright green nuclei and barely visible dull brown cytoplasm, even a single endometrial cell tended to stand out vividly (Fig. 1).

In the case of malignant endometrial cells the intensity of fluorescence and brightness of colour was found to vary from case to case and even from cell to cell, and on occasion the malignant cells were less bright than benign endometrial cells in the proliferative phase (Fig. 2). The cytoplasmic colour appeared to depend on the degree of distension of the cell. The smaller the malignant cell the redder and denser was the cytoplasm (Fig. 3). Increase in size, if caused by secretion within the cell, resulted in a relative loss of cytoplasmic brilliance. In this type of cell, the cytoplasm appeared lacy or vacuolated, the red of the cytoplasm being interspersed with green zones of secretion (Fig. 2). In the very large ballooned-out cell, there was no more than a rim of red encircling a greenish vacuole (Fig. 4).

The nucleus of the malignant cell stained yellow or greenish yellow. It frequently appeared dense and hyperchromatic, but on occasion was vesicular. The nuclear cytoplasmic ratio was grossly altered in favour of the nucleus in the smaller cell, but in a large one, the nucleus occupied a small proportion of the distended cell. The chromatin was relatively coarse and irregularly spaced (Figs. 2, 3, 4).

Large nucleoli were frequently seen but were *not* an invariable feature of the malignant cell, so much so that while they were clearly visible in some of the cells in an obviously malignant cluster, in other cells in the same cluster, they were conspicuous by their absence (Fig. 2).

A few malignant endometrial cells had dull green cytoplasm, denoting either a lack or a loss of cytoplasmic RNA. Such cells displayed other features of degeneration such as loss of distinct cell borders, foamy cytoplasm and amorphous, featureless coagulated looking nuclei. It was observed that such cells were the exception and were invariably accompanied by others displaying brilliant fluorescence and well preserved morphological details (Fig. 5).

The benign hyperplastic endometrial cells had the same quality of fluorescence and colour as small malignant cells. They displayed minimal anisocytosis. The nuclei were enlarged and yellow in colour, but the densely packed chromatin was fine, regular and evenly spread. Pin point nucleoli were a common feature (Fig. 6). In some cases the distinction between the benign and hyperplastic and the small malignant cell was difficult on morphological grounds with fluorescence microscopy. This difficulty was not resolved by studying such cells with the Papanicolaou stain (Fig. 7).

*Comparison with Papanicolaou stained cells*

Since all smears were obtained in duplicate it was possible to compare retrospectively, acridine orange stained smears with Papanicolaou stained smears. It became evident that four major difficulties are resolved by fluorescence microscopy :

1. Because of its red and yellow stain and brilliant fluorescence, the acridine orange stained endometrial cell, far from being lost in the midst of polymorphs and histiocytes as is a Papanicolaou stained cell (Fig. 8) is positively set off to advantage by the green colour of the polymorph nucleus (Fig. 9).

2. The morphology of the malignant nucleus is delineated in detail by acridine orange. A comparison of identical cells (Fig. 10 and 11) stained by the two methods suggests that the Papanicolaou stain, the alcoholic counterstains of which impart such valuable translucency to the coarse squamous cell, just does not stain the endometrial cell enough to give it body, so that many cells appear degenerate.

3. The histiocyte acquires a dull brown cytoplasm and a green nucleus with acridine orange, and is thereby easily distinguished from the endometrial cell (Fig. 12). With the Papanicolaou stain this is not always possible (Fig. 13).

4. Red blood cells not only mask nucleated cells in Papanicolaou stained smears, but make satisfactory staining impossible (Fig. 14). Since red blood cells neither fluoresce, nor interfere with the tinctorial properties of acridine orange, the appearance of malignant cells remains unaffected by the presence of blood (Fig. 15, 16, 17).

*Endocervical cells*

The larger endocervical cells displayed extreme variation in intensity of colour, ranging from dull brown to flame-orange, but the morphology of the cell made its recognition easy. The small endocervical cells had the same staining reactions as the endometrial cell and distinction between the two cell types was difficult on morphological consideration. It was found that the small endocervical cells invariably came from polypi arising in the upper end of the endocervix (Fig. 18).

---

### EXPLANATION OF PLATES

FIG. 1.—A single red and yellow benign hyperplastic endometrial cell in the midst of polymorphs. Acridine Orange. ×600.

FIG. 2.—A large cluster of malignant endometrial cells and a small cluster of benign proliferative endometrial cells. The malignant cells are less bright, are distended by secretion and infiltrated with polymorphs. Macronucleoli are absent from the majority of malignant cells. Acridine Orange. ×600.

FIG. 3.—A cluster of brilliantly fluorescing orange and yellow malignant endometrial cells. The nucleus occupies most of the smaller cell. Acridine Orange. ×600.

FIG. 4.—A group of distended malignant endometrial cells. Only a rim of bright red cytoplasm is seen and the nuclei are pushed to the periphery. Acridine Orange. ×600.

FIG. 5.—A degenerate malignant endometrial cell is seen in the centre. The cell outline is indistinct, the cytoplasm is green and the nucleus appears "coagulated". Compare staining reaction and morphology with the brightly fluorescing red and yellow malignant endometrial cell below the degenerate cell. Acridine Orange. ×600.

FIG. 6.—A group of benign hyperplastic endometrial cells. The cytoplasm has a bright orange fluorescence. The cells display minimal anisocytosis, the nuclear chromatin is normal. Pin point red staining nucleoli are present. Acridine Orange. ×600.

FIG. 7.—Hyperplastic endometrial cell diagnosed as malignant. Case of post-menopausal cystic hyperplasia. Papanicolaou. ×450.

FIG. 8.—Discrete malignant endometrial cells are not easily discerned in the midst of polymorphs and histiocytes. Papanicolaou. ×120.

FIG. 9.—Same case as in Fig. 8. The red and yellow endometrial cells stand out vividly. Acridine Orange. ×150.

FIG. 10.—A comparison of discrete malignant endometrial cells stained by Papanicolaou's method and acridine orange. The Papanicolaou stained cell has a barely visible cytoplasm and a nucleus with very little chromatin. In the AO stained cell the cytoplasm appears well preserved and the chromatin pattern is much more detailed. The AO stained cell appears larger partly because of the greater magnification and partly because it has been air-dried. Papanicolaou. ×290. Acridine Orange. ×600.

FIG. 11.—Same case as in Fig. 10. These cells are similar to the ones seen in post-menopausal atypical endometrial hyperplasia. Papanicolaou. ×290. Acridine Orange. ×600.

FIG. 12.—Histiocytes in a smear positive for adenocarcinoma. The brown and green staining distinguishes these cells from endometrial cells, even degenerate ones (cf. Fig. 5). Acridine Orange. ×600.

FIG. 13.—Same case as in Fig. 12. Some of the histiocytes are easily confused with endometrial cells (cf. Fig. 7 and 8). Papanicolaou. ×450.

FIG. 14.—A cluster of malignant cells in a blood stained smear from a case of metastatic carcinoma. The staining is unsatisfactory and the morphological features blurred. Papanicolaou. ×450.

FIG. 15.—Same case as Fig. 14. The cells stained bright red and yellow. Nuclear detail is clearly seen. Acridine Orange. ×575.

FIG. 16.—Same cases as Fig. 14 and 15. Moderately degenerate malignant cell. Enough nuclear detail remains for a correct diagnosis. Acridine Orange. ×575.

FIG. 17.—Same case as in Fig. 14, 15, 16. Section of the primary ovarian carcinoma which metastasized to the endometrium. Haematoxylin and Eosin. ×260.

FIG. 18.—A cluster of small columnar cells from an endocervical polyp mistaken for benign endometrial cells. Acridine Orange. ×600.

FIG. 19.—A cluster of malignant cells from an endocervical adenocarcinoma. Acridine Orange. ×575.

FIG. 20.—Same case as in Fig. 19. A group of malignant cells from a leiomyosarcoma. Acridine Orange. ×575.

FIG. 21.—Same case as in Fig. 3. The relatively dull cells at the periphery of the bright cluster of malignant endometrial cells display features of squamous metaplasia, namely, enlargemert, green staining cytoplasm, dense texture, intercellular bridges. Acridine Orange. ×575.

FIG. 22.—Same case as in Fig. 3 and Fig. 21. Section of endometrium showing adenoacanthoma. Haematoxylin and Eosin. ×450.

1

2

3

4

5

6

Grubb-

8

9

10

11

12

18

Grubb.

7

13

14

15

16

17

19

20

21

22

Grubb.

### RESULTS

On the basis of morphological criteria described above, an attempt was made to diagnose endometrial cancer and to distinguish it from benign hyperplasia. There were a few cases in which apparently normal endometrial cells arranged in little clusters were noted after the tenth day of the menstrual cycle or in post-menopausal women. These could not be classified, but their presence was reported.

The results of all the cases are discussed below under four headings.

*Histologically proven endometrial cancer*

Vaginal and cervical smears were studied from 29 patients who have had an endometrial carcinoma, primary or secondary, demonstrated histologically and the results of the cytological investigations are given in Table I.

TABLE I.—*Histologically Proven Endometrial Cancer*

| | |
|---|---|
| Number of cases | 29 |
| Cytology positive | 17 |
| Cytology suspicious | 5 |
| Atypical columnar cells in post-menopausal patients | 2 |
| Cytology negative | 2 |
| Unsatisfactory smears | 3 |

Of the 17 cytologically positive cases, 4 were between the ages of 44 and 48 years. Of these, one patient had a brown discharge off and on and two bouts of bleeding within a year of a normal period. The symptoms were attributed to menopause and the vaginitis she was known to have. The correct diagnosis of malignancy was primarily due to cytology. The other 3 patients were pre-menopausal. A clinical diagnosis of endometrial cancer was made in one patient at her first visit, while the other 2 were diagnosed as cases of " dysfunctional bleeding ".

Of the 5 cytologically suspicious cases, 3 were pre-menopausal, ages 42, 46 and 53 years. In all 3 patients the clinical diagnosis was dysfunctional bleeding.

The 2 patients in whom atypical columnar cells were seen were 65 and 80 years of age, the clinical diagnosis obvious, and no time was wasted in obtaining repeat smears as both patients were admitted immediately for treatment.

One of the clinically obvious cases of carcinoma with post-menopausal bleeding correctly diagnosed by cytology had two tumours, an adenocarcinoma of the endocervix and a leiomyosarcoma. The two types of malignant cells were identified in the smears and a cytological diagnosis made of the two tumours (Fig. 19 and 20).

In another patient who presented with post-menopausal bleeding a large number of cancer cells were seen singly and in clusters (Fig. 3). Attached to one of these groups and forming an integral part of it were cells which were larger in size, had a greenish cytoplasm and appeared visually to have a denser texture. A hint of intercellular bridges was apparent (Fig. 21). A cytological diagnosis of squamous metaplasia in an endometrial carcinoma was confirmed histologically (Fig. 22).

The 2 negative cases were rescreened after the histological diagnosis became known. In one, no cells recognizable as endometrial cells were detected. In the other, a few suspicious cells were seen. This patient, however, had severe atrophic vaginitis and the differences between the enlarged endometrial cells and the inflamed basal squamous cells were slight.

The three unsatisfactory smears were vaginal aspiration smears from single women and will be further discussed below.

*False positive cytology*

During this period five false positive and six false suspicious reports have been given. An analysis of these cases is given in Table II.

TABLE II.—*Analysis of False Positive Cases*

| | Patient | Age | Presenting symptoms | Histological Diagnosis |
|---|---|---|---|---|
| 1 | W. B. | 50 | Post-menopausal bleeding | Post-menopausal cystic hyperplasia. |
| 2 | D. B. | 51 | Heavy irregular uterine bleeding | Secretory hyperplasia. |
| 3 | E. D. | 54 | Irregular periods | Post-menopausal atypical hyperplasia. |
| 4 | E. E. | 68 | Post-menopausal bleeding. Lower abdominal pain. Loss of weight | Post-menopausal cystic hyperplasia. Adenomyosis. |
| 5 | F. G. | 49 | Prolonged period following amenorrhoea 9/12 | T.B. salpingitis. Pyometra. Endometrial hyperplasia. |
| 6 | Le F. | 61 | Post-menopausal bleeding | Endometrial adenomatous polyp. Haemorrhagic senile endometritis |
| 7 | V. T. | 28 | Irregular periods | Hyperplastic haemorrhagic abundantly mucus secreting endometrium. |
| 8 | E. C. | 53 | Irregular periods followed by post-menopausal spotting | No endometrium on curettings. Clinical diagnosis — menopause. To be investigated further. |
| 9 | G. F. | 44 | Heavy irregular periods | No currettings obtained on two occasions. No symptoms. Clinical diagnosis—menopause. |
| 10 | H. G. | 69 | Sciatica. Uterus deflected to one side. Referred for dilatation and curettage | Atrophic endometrium. Chronic cervicitis with hyperplasia of of cervical glands. |
| 11 | L. W. | 56 | Post-menopausal bleeding | No curettings obtained. Patient has since defaulted. |

Patient No. 4—E. E.—was discharged after an unfruitful curettage, readmitted because of the suspicious cytological report and the hysterectomy specimen revealed the adenomyosis and post-menopausal cystic hyperplasia, which would account for her symptoms.

In the case of No. 7—V. T.—it must be stated that the patient's age was believed to be 48 years at the time the cytological diagnosis of cancer was made. It is a moot point whether the necessary courage or foolhardiness would have been forthcoming had the correct age been known. The case was discussed with the clinician, and the smears repeated. Once again, hyperplastic and atypical columnar cells, probably endometrial, were seen. Three and a half months after the initial positive report, the symptoms still continuing, a curettage was done with the findings described in Table II.

Patient No. 8—E. C.—complained of three bouts of bleeding at irregular intervals after the last normal period, the longest interval being 5 months. No curettings could be obtained following the positive report. A diagnosis of menopause was considered but the patient was kept under careful observation. She now complains of " spotting ", one year after her last bout of bleeding, and is to be investigated further.

There is only one patient 9—G. F.—in this series, who has been subjected to surgery because of a cytological report. This patient was diagnosed as a case of metropathia and was put on the waiting list for a diagnostic curettage, with a note that if no endometrial polyp was found to account for her symptoms, hysterectomy might be considered. She was admitted immediately because of the positive cytology report. The very scanty curettings obtained proved insufficient for diagnosis. Subsequent smears were reported as containing hyperplastic endometrial cells. A second curettage, 6 months after the first, was totally unsuccessful. This patient has been symptom-free for over one year and is believed to be menopausal.

In this series of eleven false positives, there are, therefore, 7 patients with significant abnormalities of the endometrium which on cytological considerations could not be distinguished from a well differentiated carcinoma. In only one case, No. 10, has a careful search failed to reveal any endometrial abnormality. The simple hysterectomy performed on this patient, despite a negative curettage, was in no way influenced by the cytology report. Two cases, No. 8 and No. 11, await further investigation. One, No. 9, is assumed for the moment to be a false positive despite the lack of histological evidence, as the patient is symptom-free.

*Cytology : benign endometrial hyperplasis*

In 45 patients a cytological diagnosis of benign endometrial hyperplasia was made. This was based on the presence in the smears after the tenth day of the menstrual cycle or in post-menopausal women, of benign hyperplastic endometrial cells which have been described above. Frequently more than one type of endometrial cell could be seen, that is a mixed population of endometrial cells was noticed but none of the cells displayed any features of malignancy. An analysis of these cases is given in Table III.

There were no cases of endometrial cancer in this group.

TABLE III.—*Cytological Diagnosis, Benign Endometrial Hyperplasia*

| Histological Diagnosis | Number |
|---|---|
| Proliferative hyperplasia | 20 |
| Secretory hyperplasia | 1 |
| Post-menopausal atypical hyperplasia | 1 |
| Endometrial polyp | 2 |
| Senile endometritis | 1 |
| Incomplete abortion | 2 |
| Salpingitis | 1 |
| Menstrual endometrium | 2 |
| Normal proliferative endometrium | 1 |
| Normal secretory endometrium | 2 |
| Curettings insufficient for diagnosis | 2 |
| Cervical polyp | 2 |
| Bleeding cervical erosion | 1 |
| No follow up | 5 |
| Delayed period (clinical diagnosis) | 2 |
| | 45 |

*Normal endometrial cells. Non specific appearances*

In 5 patients benign endometrial cells arranged in little polyp-like clusters were observed and reported. These are classified in Table IV.

Cells in this group did not appear to be hyperplastic and once again no cancers were present in this group.

TABLE IV.—*Normal Endometrial Cells : Non-Specific Appearance*

| | |
|---|---|
| Secretory hyperplasia of endometrium. Fibroids . . | 1 |
| Proliferative hyperplasia of endometrium. Fibroids . | 1 |
| Normal endometrium. Fibroids . . . . . | 1 |
| Septic abortion . . . . . . . . . . | 1 |
| Cervical polyp . . . . . . . . . . | 1 |
| | 5 |

DISCUSSION

The basic features of malignant endometrial cells seen in Papanicolaou stained smears have been described in detail (Berg and Durfee, 1958 ; Koss and Durfee, 1961, 1962 ; Graham, 1963 ; Wachtel, 1964). In the present study with acridine orange fluorescence microscopy, while in general the same morphological features were observed, certain differences have been noted. Most endometrial cells, whether benign or malignant, fluoresce brightly and have an orange-red cytoplasm and yellow-green nucleus. Against the black background and in the midst of green polymorphs, the cells are seen to have a clearly defined border, and neither the morphology nor the histochemical reaction between acridine orange and the nucleic acids within the cell supports the view that the majority of exfoliated endometrial cells are degenerate.

Another point of significance resolved by the use of acridine orange is the presence or otherwise of nucleoli in malignant cells. When haematoxylin is used as a nuclear stain, it is not always possible to distinguish between a true nucleolus and a large coarse clump of chromatin. The distinction is a matter of some importance for while coarseness of chromatin is one of the features of malignancy, it may also be seen in a degenerating benign nucleus, in which the chromatin breaks up into irregular clumps possibly before karyorrhexis. With acridine orange the nucleolar RNA acquires the same orange-red colour as the cytoplasm and consequently cannot be confused with the yellow or green staining chromatin. Because of this it became apparent that the nucleolus on which so much emphasis has been laid (Koss and Durfee, 1962) is not a constant feature of malignant cells. Moreover, it is frequently present in benign hyperplastic endometrial cells, and unless the nucleolus itself is abnormal in size and shape, the distinction between a benign and malignant cell must be based on a study of the entire nucleus and its relation to the rest of the cell.

A major difficulty encountered in the cytodiagnosis of endometrial cancer is that of differentiating between the endometrial cells and a small histiocyte. According to Berg and Durfee (1958) smears from patients with endometrial cancer contain recognizable endometrial cells, recognizable histiocytes and cells which are half-way between the two. Graham (1963) emphasizes the fact that the distinction is extremely difficult unless the endometrial cell is well preserved, and goes on to add that this happens rarely. With AOFM, histiocytes have a brown staining cytoplasm and a dull green nucleus. Even the degenerate endometrial cell, which has lost the red cytoplasmic stain, has a totally different appearance.

Certain diagnostic problems remain. Cells from the upper end of the endocervix, especially when exfoliated in tight clusters, have the same staining reactions as endometrial cells. This is true also of the variety of columnar cells seen in post-coital vaginal smears. These cells, which are presumably from the male partner, display enough variation in size and appearance to raise a suspicion of endometrial cancer. Consequently no attempt is made to identify endometrial cells in post-coital smears.

The present study has shown that the identification of exfoliated endometrial cells is made considerably easier by the use of acridine orange fluorescence microscopy. The question that arises is whether any practical gain is acquired by the use of a cytological technique aimed principally at diagnosing an endometrial cancer. It is frequently argued that any woman with post-menopausal bleeding or dysfunctional bleeding will in any case undergo a diagnostic curettage. In view of the fact, however, that dysfunctional bleeding becomes increasingly common after the early forties, and that most hospital patients so diagnosed have their names put on a " waiting list ", a cytological diagnosis of endometrial cancer will result in some of these patients " jumping the queue ". In the small series of 29 cases of endometrial cancer reported here, there were 7 patients who were pre-menopausal. In only one of these was a diagnosis of cancer made on clinical grounds. It is not intended to suggest that a cytological diagnosis is a substitute for a histological one, nor that a negative cytological report should preclude a diagnostic curettage.

When the purpose of a laboratory investigation is to direct attention to an unsuspected lesion which will subsequently be investigated further, a false positive report ceases to be of great significance. In the detection of endometrial cancer, however, an excessively large number of such reports would defeat this purpose, namely that of selecting patients who must have an immediate curettage. In a period of 3 years nine such reports have been given. Only one patient in this series has been shown to have an endometrium normal for her age, while in another, it was the false positive cytology report, which, despite the negative curettage, led to a therapeutic hysterectomy. That benign but pathological endometrial cells are not always confused with malignant ones is demonstrated by the 50 cases listed in Tables III and IV.

One question remains. What type of specimen is suitable and should be used. The majority of reports on the cytodiagnosis of endometrial cancer categorically state that the vaginal smear is to be preferred to a cervical scrape. In the present series there were 5 patients in whose vaginal smears few or no cancer cells were seen, while the cervical smears were full of malignant cells. In at least 3 of these cases the number and variety of cells in the vaginal smears were insufficient for diagnosis. There was no case in which the cervical smear did not contain enough cells for diagnosis, though there were many in which the vaginal smears contained a greater number of cells. The 3 false negative cases reported above were single women, and vaginal aspiration smears only were received from these patients. This divergence from the generally accepted method led to a closer evaluation of smears from cases of endometrial cancers. It was noticed that the vaginal smear contained more endometrial cells if the patient was bleeding at the time that the specimen was collected, whereas those patients in whom the cervical smear was the better of the two gave a history of abnormal bleeding, but did not have overt bleeding at the time the specimen was collected. It is likely that cells from an endometrial cancer are caught in the cervical mucus plug, and appear in the vagina only when the mucus plug is mechanically dislodged by haemorrhage. If this hypothesis is correct the cervical smear may prove to be important in diagnosing early endometrial cancer in a woman with minimal symptoms. This would make it unnecessary for laboratories which screen only cervical smears to double their work in the search for endometrial cells. When the patient presents with overt bleeding an additional vaginal smear is unlikely to be beyond the scope of even the busiest laboratory.

### SUMMARY

Acridine orange fluorescence microscopy (AOFM) has been used over a period of three years to study endometrial cells exfoliated into vaginal and cervical smears.

The appearances of malignant and benign hyperplastic endometrial cells are described.

The smears have been compared with the corresponding Papanicolaou smears and the advantages of using AOFM for endometrial cytology are discussed and illustrated.

Of 29 cases of endometrial cancer screened by AOFM, 17 were reported as positive and 5 as suspicious. Two cases had abnormal columnar cells, 2 were reported negative and 3 had unsatisfactory smears.

Eleven false positive reports are discussed. Two of these cases await further investigation, and only one had a normal endometrium.

The role of exfoliative cytology in the detection of endometrial cancer is considered and the nature of the specimen required for this purpose is discussed.

I am grateful to the British Empire Cancer Campaign for Research for supporting this study with a research grant and to Professor K. R. Hill for his supervision of the work, half of which formed part of a thesis.

I also wish to thank Mrs. A. Kemplay for her able technical assistance, Mr. R. R. Phillips for the black and white prints and Miss I. Goldapple for work on the typescript.

### REFERENCES

AYRE, J. E.—(1951) ‘ Cancer Cytology of the Uterus. Introducing a new concept of cervical cell pathology ’. New York (Grune & Stratton).

BERG, J. W. AND DURFEE, G. R.—(1958) *Cancer, N.Y.*, **11**, 158.

VON BERTALANFFY, L., MASIN, F. AND MASIN, M.—(1956) *Science, N.Y.*, **124**, 1024.

VON BERTALANFFY, L., MASIN, F. AND MASIN, M.—(1958) *Cancer, N.Y.*, **11**, 873.

BODDINGTON, M. M. AND SPRIGGS, A. I.—(1965) *Br. med. J.*, i, 1523.

DE BRUX, J. A.—(1958) *Acta cytol.*, **2**, 538.

ELEVITCH, F. R. AND BRUNSON, J. G.—(1961) *Surgery Gynec. Obstet.*, **112**, 2.

GRAHAM, R. M.—(1963) ‘ The Cytologic Diagnosis of Cancer.’ Philadelphia & London (W. B. Saunders & Co.).

GRUBB, C. AND CRABBE, J. G. S.—(1961) *Br. J. Cancer*, **15**, 483.

VON HAAM, E.—(1958) *Acta cytol.*, **2**, 580.

HAMPERL, H. AND SCHÜMMELFEDER, N.—(1961) *Ciba-Symposium*, **9**, 50.

HORAVA, A., DE NEEF, J. C., BOUTSELIS, J. C. AND VON HAAM, E.—(1961) *Clin. Obstet. Gynec.*, **4**, 1128.

JORDAN, M. J., BADER, G. M. AND NEMAZIE, A. S.—(1956) *Obstet. Gynec. N.Y.*, **7**, 646.

Koss, L. G. and Durfee, G. R.—(1961) 'Diagnostic Cytology and its Histopathologic Basis'. London (Pitman Medical Publishing Co. Ltd.).
Koss, L. G. and Durfee, G. R.—(1962) *Acta cytol.*, **6**, 519.
McLaren, H. C.—(1963) 'The Prevention of Cervical Cancer'. London (The English Universities Press Ltd.).
Marks, R. and Goodwin, A. M.—(1962) *Br. J. Cancer*, **16**, 390.
Mezzadra, J. M. and Terzano, G.—(1958) *Acta cytol.*, **2**, 539.
Papanicolaou, G. N. and Traut, H. F.—(1943) 'Diagnosis of Uterine Cancer by Vaginal Smear'. New York (The Commonewealth Fund).
Pundel, J. P.—(1958) *Acta cytol.*, **2**, 588.
Sani, G., Citti, U. and Caramazza, G.—(1962) *Minerva ginec.*, **14**, 389.
Varangot, J., Granjon, A., Nuovo, V. and Vassy, S.—(1954) *Am. J. Obstet. Gynec.*, **68**, 474.
Wachtel, E.—(1958) *Acta cytol.*, **2**, 538.
Wachtel, E.—(1964) 'Exfoliative Cytology in Gynaecological Practice'. London (Butterworths).